# Supplementary figures and images for: Oxytocin neurons drive melanocortin circuit maturation via vesicle release during a neonatal critical period
Source: PLoS Biol. 2025 Nov 12;23(11):e3003158. doi: 10.1371/journal.pbio.3003158 (PMC12633931; doi:10.1371/journal.pbio.3003158)

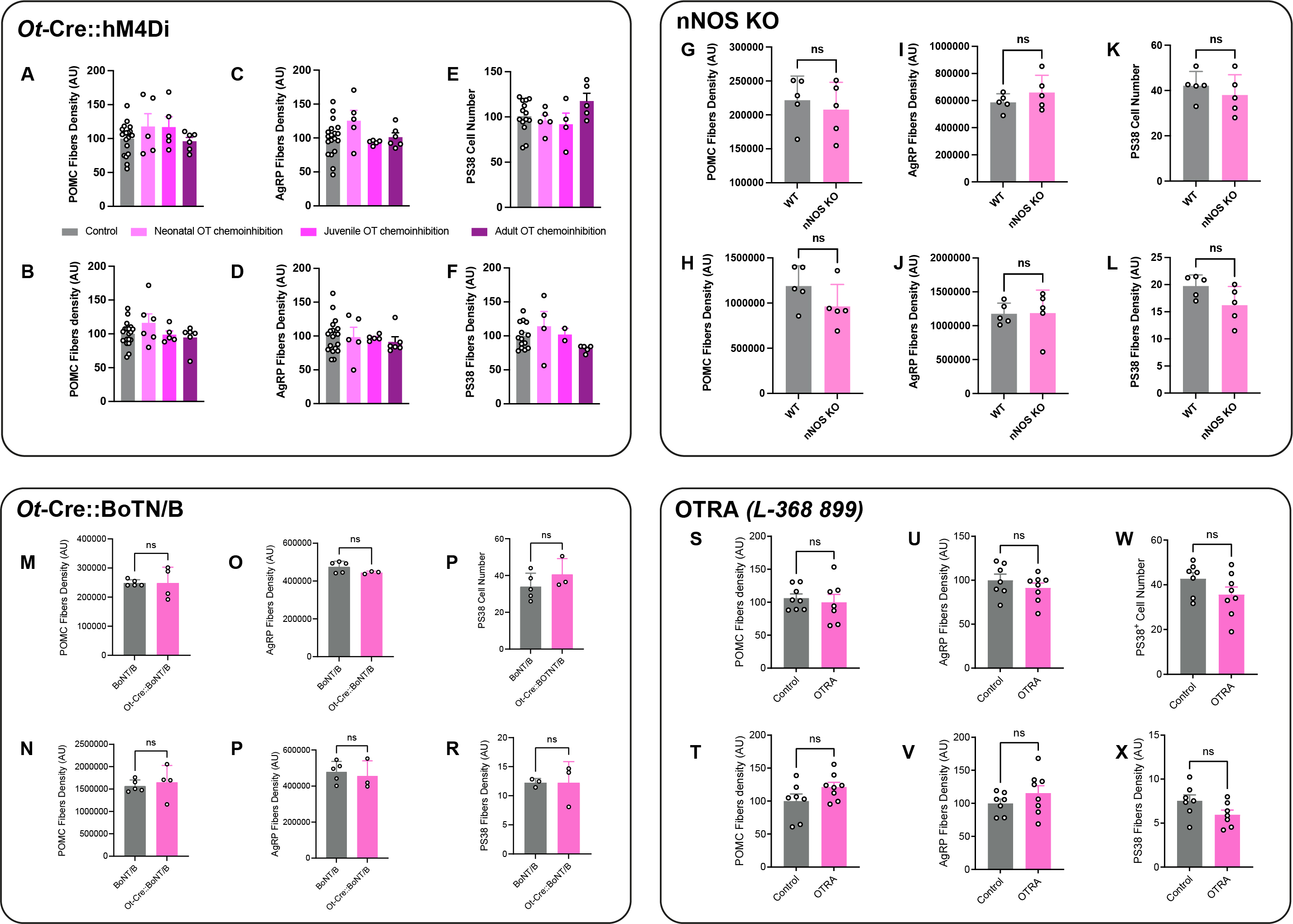

Supplement: S1 Fig — Quantification of the density of (A, B, G, H, M, N, S, T) POMC and (C, D, I, J, O, P, U, V) AgRP fibers in the (A, C, G, I, M, O, S, U) PVH, and (B, D, H, J, N, P, T, V) DMH and quantification of (E, K, P, W) the number of OT neurons in the PVH and (F, L, R, X) the density of OT fibers in the LHA of adult control and (A–F) Ot-Cre::R26-LSL-hM4Di-DREADD female mice injected with Compound-21 neonatally, or during juvenile life, or during adulthood (n = 5–7 animals per group), (G–L) nNOS KO female mice (n = 5 animals per group), (M–R) Ot-Cre::BoNT/B adult female mice (n = 5–3 animals per group), or (S–X) female mice injected with the OTR antagonist L-368,899 neonatally (n = 7–8 animals per group). Data are presented as means + SEM. The data underlying this Figure can be found in S1 Data (JPG) [file pbio.3003158.s002.jpg]

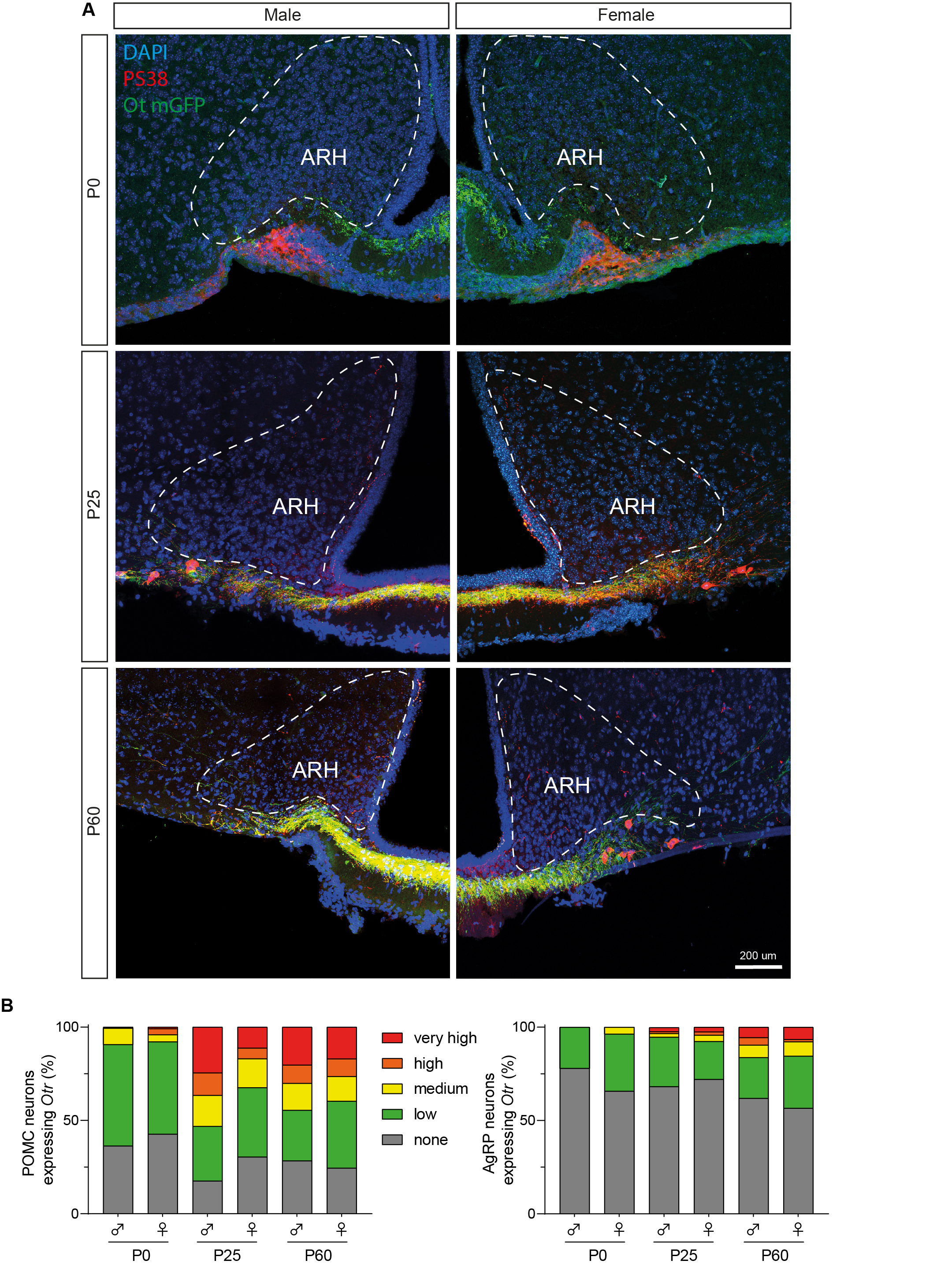

Supplement: S2 Fig — (A) Representative images of OT fibers innervating the arcuate nucleus (ARH) of P0, P25, and P60 male and female mice as revealed by an immunostaining against oxytocin (PS38 antibody) and genetic labeling of OT fibers (Ot mGFP mice). (B) Quantification of Otr co-expression in Pomc and Agrp neurons of P0, P25, and P60 male and female mice (n = 3–4 animals per group). Scale bar, 200 μm. The data underlying this Figure can be found in S1 Data (JPG) [file pbio.3003158.s003.jpg]

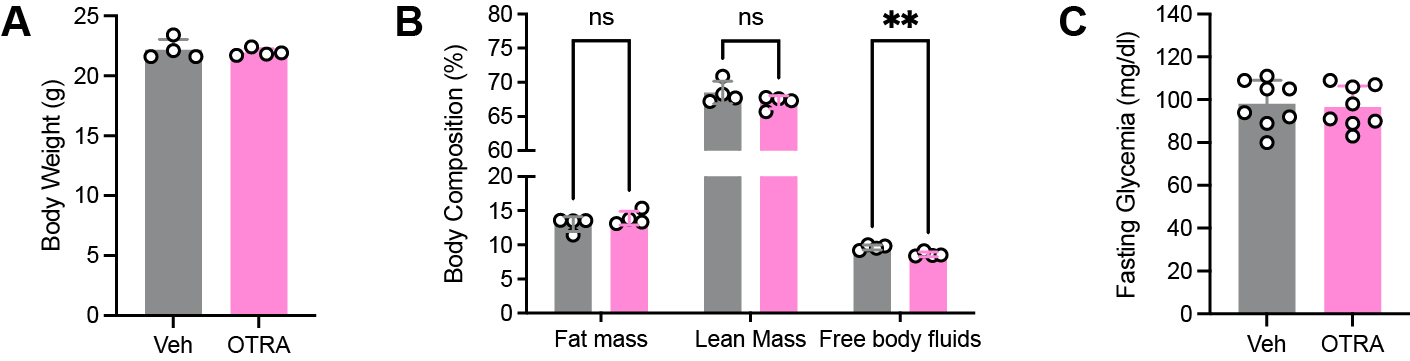

Supplement: S3 Fig — (A) Body weight, (B) body composition, and (C) fasting glycemia, in adult female mice injected with saline (control) or an oxytocin receptor antagonist (L-368,899, OTRA) neonatally (n = 4–8 animals per group). Data are presented as means ± SEM. **P < 0.01. The data underlying this Figure can be found in S1 Data (JPG) [file pbio.3003158.s004.jpg]
