# Supplementary material for: Oxytocin neurons drive melanocortin circuit maturation via vesicle release during a neonatal critical period
Source: PLoS Biol. 2025 Nov 12;23(11):e3003158. doi: 10.1371/journal.pbio.3003158 (PMC12633931; doi:10.1371/journal.pbio.3003158)
Supplement: Attachment — Submitted filename: ResponseReviewers.pdf [file pbio.3003158.s007.pdf]

We thank the Reviewers for their thoughtful treatment of our manuscript and their constructive comments. The manuscript has been revised according to these comments including new experimental data (Figure 5A-B, Figure 9A, new Supplementary Figure 2).

Response to Reviewer #1's comments

Comment 1. "*Mechanism of OT Action Remains Vague-- Although OT is shown to act in a paracrine manner and OTRs are present in ARH neurons, the site of OT release remains speculative. The lack of a loss-of-function model specific to the OT peptide (as opposed to neuron inhibition or SNARE-mediated exocytosis) makes it difficult to attribute the phenotype solely to OT rather than other co-released factors. Discussion should be expanded to include these limitations of the study.*"

Response. We agree with the reviewer that, although our study provides compelling evidence that oxytocin neurons play an important role in the development of the melanocortin system, it does not definitively demonstrate that oxytocin release per se acts via oxytocin receptors (OTRs) to promote axonal development of POMC and AgRP neurons. Addressing this question would indeed require loss-of-function models such as inducible POMC- and AgRP-specific OTR knockouts. However, generating and characterizing such models would entail significant time and effort, which would substantially delay the publication of the present work. Nevertheless, our data support the hypothesis that oxytocin acts, at least in part, directly on POMC neurons to influence the development of their projections. First, the chemogenetic inhibition of OT neurons during neonatal life disrupts POMC and AgRP neural projections. Second, our *ex vivo* arcuate nucleus explant experiments show that direct exposure to oxytocin promotes axonal growth. Third, neonatal administration of an oxytocin receptor antagonist disrupts the development of POMC projections in vivo. The mechanism by which oxytocin neurons influence AgRP projections remains to be fully elucidated. However, our findings using *Ot*-Cre::BoNT mice suggest that this may involve the co-release of other neurotransmitters or neuropeptides from oxytocin neurons, such as glutamate or vasopressin, both of which have been shown to influence axon growth in other neuronal systems (PMID: 2889618, 10500228, 8077964, 11068133). These points have been addressed in the revised Discussion section (p. 11, ln. 18-27 and p. 12, ln. 1-3 and ln. 17-21).

Comment 2. "*AgRP Circuit Inconsistencies-- OT manipulations affect both POMC and AgRP neurons in some experiments but not consistently across all conditions (e.g., OTRA affects only POMC fibers). This partial effect requires mechanistic discussion, particularly since AgRP neurons express OTRs (though to a lesser extent). While the study is already robust, demonstrating the localization of OT fiber density in the ARH during early postnatal life would provide valuable information to validate the hypothesis. In the discussion, it is noted that the ARH is virtually devoid of OT fibers (Barelle & Bouret, unpublished observation). Were these observations made in young animals? It is possible that OT innervation is stronger during early development, which could account for the neurotrophic effects of OT*"

Response. We agree that the lack of effect of oxytocin receptor antagonist (OTRA) on AgRP projections may seem unexpected. However, the proportion of AgRP neurons expressing OTR appears relatively modest, which supports the idea that oxytocin neurons may influence AgRP projections via the release of other factors. Our findings using Ot-Cre::BoNT mice suggest that this effect may involve the co-release of additional neurotransmitters or neuropeptides from OT neurons, such as glutamate or vasopressin, both of which have been shown to modulate axon growth in other neuronal systems (PMID: 2889618, 10500228, 8077964, 11068133). We included this point in the revised Discussion (p. 11, ln. 26-27, and p. 12 ln. 1-3).

Regarding the absence of detectable oxytocin fibers in the arcuate nucleus, this observation was consistent across multiple developmental stages, including P0, P25, and P60. We agree with the reviewer that this is an important finding and have now included it in Figure 5.

Comment 3. "*Sex Differences Underexplored-- The striking male-specific effects are presented but not mechanistically addressed. The authors could speculate whether hormonal differences or OT/OTR expression levels contribute*."

Response. We appreciate the reviewer's insightful comment on the intriguing sex differences observed in the effects of oxytocin on the development of the melanocortin system. This point was also raised by Reviewers 2 and 4. Several potential explanations could account for the distinct impacts of oxytocin on the development of POMC and AgRP neurons in males and females. One hypothesis involves that the expression of *Otr* in POMC/AgRP neurons may be sexually dimorphic. We investigated this by examining the density of *Otr* mRNA expression in POMC and AgRP neurons of both male and female mice, including during neonatal life, but found no differences (new Supplementary Figure 2). Additionally, there could be inherent differences in oxytocin projections to the arcuate nucleus between males and females. To explore this, we analyzed oxytocin projections using immunohistochemistry and genetic labeling of oxytocin axons but did not find any apparent sex differences in the density of oxytocin terminals in the arcuate nucleus (new Supplementary Figure 2). Therefore, the marked sexual dimorphism in the effect of oxytocin on the development of the melanocortin system remains unexplained but could involve more other mechanisms or factors, such as sex hormones, including estrogens that have been shown to have protective effects on metabolic regulations (PMID: 23460719). This information, including experimental data on *Otr* expression and oxytocin terminals in the arcuate nucleus, has been added in the revised manuscript (Results, p. 8, ln. 6-7 and ln. 21-22; Discussion, p. 12, ln. 22-27 and p. 13, ln. 1-5; new Supplementary Figure 2).

Comment 4. "*Minor Issues by Figure*
Figure 5
Panel 5C indicates ARC instead of ARH
Response. We changed ARC to ARH

Figure 7, Figure 8 and Suppl Fig 1
The figure legend refers to "L-368,899," but the compound is called "L-368,889" in the rest of the manuscript. This is likely a typo and should be corrected for consistency."
Response. We apologize for the typo. We corrected the name of the antagonist to L-368-899 throughout the manuscript and Figures

Response to Reviewer #2's comments

Comment 1. "*Could the authors provide more detail on the developmental timeline of POMC and AgRP axon innervation into the PVH relative to the appearance of OT neurons/fibers there (perhaps referencing Figure 2 or adding supplemental information)? This context is crucial to determine whether the neonatal chemogenetic inhibition of OT neurons affects the initial targeting and innervation by POMC/AgRP axons or the subsequent maintenance of these connections*."

Response. We thank the Reviewer for raising this excellent point. The development of POMC and AgRP projections has been extensively studied. Prior work indicates that these neural circuits develop primarily during the second postnatal week in mice, with innervation of the paraventricular nucleus occurring between postnatal day (P) 8 and P10 (PMID: 15028773; PMID: 12559095; PMID: 15804403). In contrast, the ontogeny of oxytocin (OT) neurons and their projections has been less thoroughly characterized, though several studies have addressed this question (PMID: 25767437; PMID: 29044774; https://doi.org/10.1016/0196-9781(80)90101-1). To further investigate this, we analyzed OT neuronal projections using both immunohistochemistry and genetic labeling of OT neuron membranes at the ages when chemogenetic manipulations were performed (*i.e.*, P0, P25, and P60). Data show that OT cell

bodies are present in the PVH as early as P0, exhibiting short processes at this stage that progressively extend during the first two postnatal weeks. These findings support the hypothesis that OT neurons may play a more prominent role in the initial targeting or formation of POMC/AgRP innervation, rather than in the maintenance of these connections later in life. We have included this information in the Results (p. 7, ln. 24-26, p. 8, ln. 1-7) and Discussion sections of the revised manuscript (p. 12, ln. 5-7), and we now present the immunohistochemical and genetic-labeling data in the new Figure 5A.

Comment 2. "*The study does not definitively establish a direct, cell-specific role for OT signaling on the target neurons (POMC/AgRP). While the current data are suggestive, utilizing conditional OTR knockout mice would be required to unequivocally demonstrate that OT acts directly on these populations to guide their projections. This limitation should be acknowledged in the Discussion*."

Response. We thank the reviewer for her/his valuable comment, which was also raised to some extent by Reviewer 1. Although our study provides compelling evidence that oxytocin neurons play an important role in the development of the melanocortin system, we agree that it does not definitively demonstrate that oxytocin directly acts via OTR to promote axonal development of POMC and AgRP neurons. Addressing this question would indeed require loss-of-function models such as inducible POMC- and AgRP-specific OTR knockouts. However, generating and characterizing such models would entail significant time and effort, which would substantially delay the publication of the present work. This limitation of our study has been included in the revised Discussion (p. 11, ln. 18-27 and p. 12, ln. 1-3 and ln. 17-21).

Comment 3. "*The possibility that OT neurons might co-release other relevant axon guidance molecules during this developmental window, which could contribute to the observed effects independently of, or in addition to, OT peptide signaling, has not been ruled out. This alternative possibility should also be considered in the Discussion*."

Response. We agree with the reviewer that oxytocin neurons may not only secrete oxytocin but also co-release other axon guidance cues that could influence the development of melanocortin circuits. In support of this idea, we have previously shown that PVH neurons secrete class 3 semaphorins, which play a critical role in the development of POMC circuits (PMID: 30661757). To further address the reviewer's suggestion, we examined the expression of class 3 semaphorins in OT neurons using the HypoMap single-cell RNA sequencing database. We found that Sema3A, Sema3C and Sema3E are enriched in OT neurons (see data below). These findings support the possibility that OT neurons may influence melanocortin circuit development through semaphorin signaling as well. We have now included these points in the revised Discussion (p. 12, ln. 9-15).

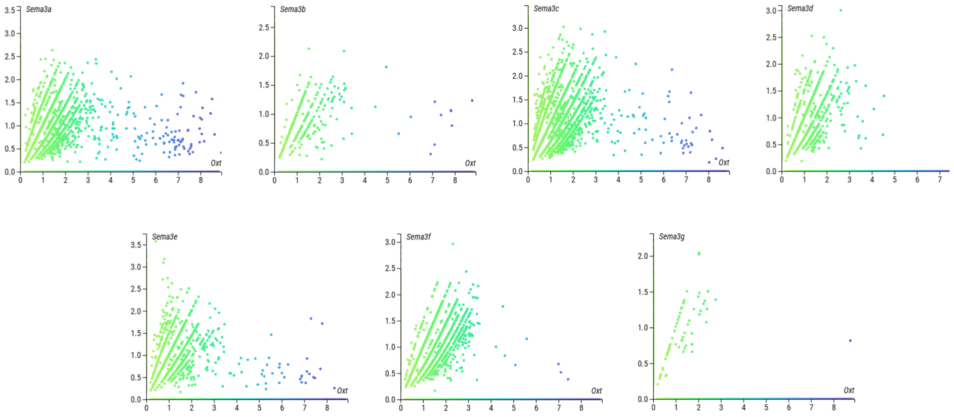

Comment 4. "*The observed sex differences, particularly the noted lack of effect in females for some manipulations, are intriguing but currently under-explored in the Discussion*."

Response. We agree with the reviewer that this observation should be discussed, as also suggested by Reviewers 1 and 4. Several potential explanations could account for the distinct impacts of oxytocin on the development of POMC and AgRP neurons in males and females. One hypothesis involves that the expression of *Otr* in POMC/AgRP neurons may be sexually dimorphic. We investigated this by examining the density of *Otr* mRNA expression in POMC and AgRP neurons of both male and female mice, including during neonatal life, but found no differences (new Supplementary Figure 2). Additionally, there could be inherent differences in oxytocin projections to the arcuate nucleus between males and females. To explore this, we analyzed oxytocin projections using immunohistochemistry and genetic labeling of oxytocin axons but did not find any apparent sex differences in the density of oxytocin terminals in the arcuate nucleus (new Supplementary Figure 2). Therefore, the marked sexual dimorphism in the effect of oxytocin on the development of the melanocortin system remains unexplained but could involve more other mechanisms or factors, such as sex hormones, including estrogens that have been shown to have protective effects on metabolic regulations (PMID: 23460719). This information, including experimental data on *Otr* expression and oxytocin terminals in the arcuate nucleus, has been added in the revised manuscript (Results, p. 8, ln. 6-7 and ln. 21-22; Discussion, p. 12, ln. 22-27 and p. 13, ln. 1-5; new Supplementary Figure 2).

Other Comments. "** Figure Legends: While comprehensive, the figure legends could potentially be condensed for better readability, perhaps by moving extensive methodological details to the main Methods section.*
** Methods Clarity: The Methods section should specify the concentration of OT used in the explant cultures and include justification for its physiological relevance.*
** Statistical Reporting: Regarding statistics, please clarify how normality was assessed prior to choosing non-parametric tests and confirm whether appropriate multiple-comparison corrections were applied where necessary*."

Response

We apologize if the figure legends were overly dense. In response, we have revised them to improve clarity and readability.

The concentration of oxytocin used in the explant assay was 100 ng/ml. We have now included this information in the Methods section (p. 20, ln. 6) and Figure Legend.

Non-parametric tests were selected in cases where the sample size was small ($n < 8$), in accordance with standard recommendations. For groups with $n \geq 8$, normality was assessed using the Shapiro–Wilk test. When the assumption of normality was violated ($p < 0.05$), non-parametric tests were used. Additionally, Dunn's test for non-parametric comparisons was applied and this is now clearly stated in the revised Methods section.

Response to Reviewer #3's comments

Comment 1. "*Pictures in Fig. 2C-2D, Fig.3B-3C and Fig.7B do not seem consistent with the conclusion shown in the statistical analysis. These pictures are suggested to be replaced to avoid unnecessary confusion. Importantly, given the importance of the validity of this comparison, it is suggested that the authors show a series of PVH sections to show a convincing comparison between the two conditions*."

Response. We apologize if the micrographs originally presented in Figure 2C-2D, Figure 3B-3C and Figure 7B were not fully representative of the quantitative data. We have now replaced it with images that more accurately reflect the results shown in the corresponding graphs.

Comment 2. “*There seems to have some inconsistency between neuron activity changes and oxtyocin receptoer activation on AgRP projections. This inconsistency needs to be clarified.*”

Response. We thank the reviewer for this insightful comment, which was also raised to some extent by Reviewers 1 and 2. The mechanism by which oxytocin (OT) neurons influence AgRP projections remains unclear. While neonatal administration of an oxytocin receptor antagonist disrupts the development of POMC projections, it does not appear to affect AgRP projections. However, our findings using Ot-Cre::BoNT mice suggest that OT neurons may influence AgRP projections through the co-release of other neurotransmitters or neuropeptides, such as glutamate or vasopressin, both of which have been shown to promote axon growth in other neuronal systems (PMID: 2889618, 10500228, 8077964, 11068133). These points have been incorporated into the revised Discussion section (p. 11, ln. 26-27, and p. 12 ln. 1-3)

Comment 3. “*Fig. 9 demonstrated the effect on Magel2KO phenotypes. The data on the KO as well as Gq control should be provided.*”

Response. We thank the reviewer for this insightful suggestion. We agree that analyzing POMC and AgRP projections in WT Ot-Cre::R26-hM3Dq-DREADD mice would be interesting. However, we currently do not have tissue available for this analysis, and regenerating the Ot-Cre::R26-hM3Dq-DREADD mouse line would require significant time and resources, which would substantially delay the completion and publication of the present study. Nonetheless, we have now acknowledged this limitation in the Discussion (p. 14, ln. 20-21).

Response to Reviewer #4’s comments

Comment 1. “*Did the authors validate that Cmpd-21 reduced activity of oxytocin neurons in hM4Di expressing mice? It is quite difficult to chronically inhibit neurons with DREADDs and some evidence should be provided to show that the DREADD inhibition worked. I don't think this is a major concern given that a similar phenotype was shown with the iBot experiment (a different form of inhibition) but this data should be provided if possible.*”

Response. We agree with the reviewer that demonstrating the effect of Cmpd-21 on the neuronal activity of oxytocin neurons in our Ot-Cre::R26-LSL-hM4Di-DREADD model is important. These validation experiments have already been conducted and are included in a separate manuscript on the role of oxytocin in heart rate and breathing, which is currently in press in *Nature Neuroscience* and available in BioRxiv (https://doi.org/10.1101/2023.09.26.559512) (see data below). We now cite this study in the revised manuscript (p. 6, ln. 2-4).

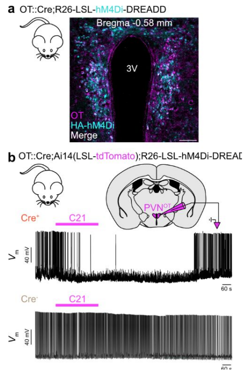

Comment 2. “*Likewise is there any validation of the chemogenetic activation (i.e. increased fos in Oxt neurons)*?”

Response. We also validated the chemogenetic activation of the *Ot*-Cre::R26-hM3Dq-DREADD mouse model by confirming that administration of Cmpd-21 induces c-Fos

immunoreactivity in oxytocin neurons. These results are now included in the revised manuscript as Figure 9A (Results, p. 10, ln. 14-16).

Comment 3. "*Do the authors have any potential explanation for the male specific effects of oxt neuron inhibition? Some discussion of this in the discussion section seems warra*nted."

Response. Several potential explanations could account for the distinct impacts of oxytocin on the development of POMC and AgRP neurons in males and females. One hypothesis involves that the expression of *Otr* in POMC/AgRP neurons may be sexually dimorphic. We investigated this by examining the density of *Otr* mRNA expression in POMC and AgRP neurons of both male and female mice, including during neonatal life, but found no differences (new Supplementary Figure 2). Additionally, there could be inherent differences in oxytocin projections to the arcuate nucleus between males and females. To explore this, we analyzed oxytocin projections using immunohistochemistry and genetic labeling of oxytocin axons but did not find any apparent sex differences in the density of oxytocin terminals in the arcuate nucleus (new Supplementary Figure 2). Therefore, the marked sexual dimorphism in the effect of oxytocin on the development of the melanocortin system remains unexplained but could involve more other mechanisms or factors, such as sex hormones, including estrogens that have been shown to have protective effects on metabolic regulations (PMID: 23460719). This information, including experimental data on *Otr* expression and oxytocin terminals in the arcuate nucleus, has been added in the revised manuscript (Results, p. 8, ln. 6-7 and ln. 21-22; Discussion, p. 12, ln. 22-27 and p. 13, ln. 1-5; new Supplementary Figure 2).

Comment 4. "*Do the authors have any explanation for the lack of effect on AgRP projections with the antagonist, but not with the chemogenetic bottox approaches?*"

Response. We agree that the lack of effect of oxytocin receptor antagonist (OTRA) on AgRP projections may seem unexpected. However, the proportion of AgRP neurons expressing OTR appears relatively modest, which supports the idea that oxytocin neurons may influence AgRP projections via the release of other factors. Our findings using Ot-Cre::BoNT mice suggest that this effect may involve the co-release of additional neurotransmitters or neuropeptides from OT neurons, such as glutamate or vasopressin, both of which have been shown to modulate axon growth in other neuronal systems (PMID: 2889618, 10500228, 8077964, 11068133). We included this point in the revised Discussion (p. 11, ln. 26-27, and p. 12 ln. 1-3).

Comment 5. "*The metabolic phenotype resulting from Oxt inhibition is hard to understand. How do the authors explain the lack of a change in body weight given increased feeding and no change in energy expenditure? It is also perplexing that a phenotype that impairs melanocortin pathways leads to measures suggesting improved metabolism (i.e. lower adiposity, elevated lean mass, improved glucose tolerance). What is the physiological relevance of this pathway for circuit development given that disrupting it improves metabolism?*"

Response. We agree that the metabolic outcomes following neonatal oxytocin receptor antagonism (OTRA) may appear paradoxical. However, oxytocin is known to act on a variety of neuronal systems, both within and outside the hypothalamus, to regulate energy metabolism (PMCID: PMC3603288; PMID: 31657509). This raises the possibility that OTRA administration during the neonatal period may have affected the development of multiple neuronal populations beyond POMC neurons, contributing to the complexity of the observed metabolic phenotype. Supporting this hypothesis, our *ex vivo* explant assay indicates that oxytocin promotes the overall neurite outgrowth of arcuate neurons (Figure 6), suggesting a broader role in hypothalamic circuit development. In addition, our RNAscope analysis shows that *Otr* is widely expressed throughout the neonatal hypothalamus, with strong expression in the ventromedial and dorsomedial nuclei (Figure 5), further highlighting potential additional sites of OTRA action during development. There is also the possibility that the oxytocin antagonist may have influenced peripheral systems involved in glucose and energy homeostasis. Notably,

Otr is highly expressed in adipose tissue (PMID: 31843930), suggesting a possible direct effect of OTRA on adipocyte development and function. We have now incorporated this discussion into the revised manuscript (p. 13, ln. 15-27 and p. 14, ln. 1-4).

Comment 6. "*What is the effect of OT neuron stimulation in WT mice? Is the increase in POMC specific to the Magel2 KO model?*"

Response. We agree that analyzing POMC and AgRP projections in WT Ot-Cre::R26-hM3Dq-DREADD mice would be interesting. However, we currently do not have tissue available for this analysis, and regenerating the Ot-Cre::R26-hM3Dq-DREADD mouse line would require significant time and resources, which would substantially delay the completion and publication of the present study. Nonetheless, we have now acknowledged this limitation in the Discussion (p. 14, ln. 20-21).

Comment 7. "*How does the explant experiment relate to AgRP/POMC projections? I assume the fiber growth in this model is not specific to just these neurons. Does this suggest that Oxt may regulate many other arcuate projections to PVN, and if so do you see changes in other arcuate projections to PVN following blockade of Oxt? Perhaps this may help to explain the complicated metabolic phenotype?*"

Response. We thank the reviewer for raising this interesting and important point. In our *ex vivo* assay, we assessed the overall axonal outgrowth of arcuate-derived neurons without distinguishing between specific subtypes. However, single-cell RNA sequencing studies (e.g., Campbell et al., Nature Neuroscience 20:484-496, 2017) have revealed that the arcuate nucleus contains up to 34 distinct neuronal populations, with only a minority expressing Agrp (~2 clusters) or Pomc (~3 clusters), representing approximately 15% of the total population. It is therefore likely that oxytocin exerts a stimulatory effect on axon growth from a broader range of arcuate neurons, potentially including POMC neurons, but also other, non-identified subtypes. This could indeed help explain the complex metabolic phenotype observed, as the reviewer suggests. Unfortunately, it is technically impossible to selectively label POMC and AgRP axons in our *ex vivo* system, due to the rapid release of these neuropeptides under culture conditions. We have now included this clarification in the revised Discussion (p. 13, ln. 15-21).

Comment 8. "*Is representative image in 4E for iBot representative? It looks like the POMC signal is much lower in iBot group.*"

Response. We apologize if the micrograph originally presented in Figure 4E was not fully representative of the quantitative data. We have now replaced it with an image that more accurately reflects the results shown in the corresponding graphs.
